# Editorial for the Special Issue “Molecular Biomarkers in Colorectal Adenocarcinoma”

**DOI:** 10.3390/ijms22042052

**Published:** 2021-02-19

**Authors:** Pinelopi I. Artemaki, Christos K. Kontos

**Affiliations:** Department of Biochemistry and Molecular Biology, Faculty of Biology, National and Kapodistrian University of Athens, 15701 Athens, Greece; partemaki@biol.uoa.gr

Colorectal cancer (CRC) is one of the most common malignancies, with an elevated mortality rate. Despite the great progress that has been achieved towards the elucidation of its molecular background, there is an imperative need for new molecular biomarkers in CRC since this malignancy is characterized by great heterogeneity. In this thematic issue, “Molecular Biomarkers in Colorectal Adenocarcinoma”, we summarize the existing knowledge in this field, adding formerly unknown perspectives. We have assembled 13 articles from prominent authors in the field, which analyze the biomarker utility of a variety of distinct molecules ranging from messenger RNAs (mRNAs) and circular RNAs (circRNAs) to components of classical signaling pathways.

The pathogenesis of CRC is considered as a multistage process characterized by somatic gene mutations, and is closely linked to the deregulation of signaling pathways critically implicated in the maintenance of homeostasis of epithelial integrity in the intestine. A growing number of studies has underscored the crucial impact of members of the tripartite motif (TRIM) protein family on most malignancies including CRC. A thorough analysis of the oncogenic signaling processes targeted by different TRIMs and their key role in the development of CRC is presented in the review article by Eberhardt et al. [1]. Additionally, in this review, it is highlighted that an in-depth understanding of the crosstalk of TRIMs with the key signaling pathways in CRC development and progression is a critical requirement regarding the validation of TRIM proteins as novel biomarkers and as potential therapeutic targets for CRC.

The poor prognosis of patients with TNM stage II and III tumors, the high potential of metastasis and the resistance to conventional therapies constitute the greatest challenges in CRC. Additionally, the absence of effective, non-invasive screening tests in the clinical practice hampers the early diagnosis of CRC. Several research studies have focused on the molecular background which could account for metastasis. An interesting article, written by Filip et al., effectively summarizes the existing literature regarding CRC distant metastasis, designating potential molecular and clinicopathological biomarkers which could possess prognostic and predictive information for CRC patients with distant metastasis [2].

Aiming to decipher the molecular basis of metastasis, the mutational status of metastatic CRC (mCRC) patients has been analyzed; it was revealed that up to 12% of mCRC patients harbor mutations in the *BRAF* gene. The most prominent one is BRAF^V600E^, which is a well-known unfavorable prognostic marker. The currently approved treatments for *BRAF*-mutated mCRC patients are characterized by low impact, while there is no established optimal treatment option. In the review article of Djanani et al., both molecular and clinical aspects of *BRAF*-mutated mCRC patients are meticulously presented to the readers, accompanied by an update on current and future treatment strategies, which may direct the therapy of mCRC in a new era [3].

As first-line treatment for mCRC, irinotecan or oxaliplatin in combination with 5-fluorouracil (5-FU) are administered. However, patients’ response rates are approximately 50%, implying that a substantial group of mCRC patients is characterized as chemo-resistant. In this context, Palshof et al. investigated the expression levels of ABCG2 transporter, the overexpression of which has been associated with multidrug resistance in various malignancies, as a predictive indicator for irinotecan therapy administered to mCRC patients [4]. Interestingly, it was shown that the ABCG2 drug efflux pump could be used as a predictive biomarker, even though further studies are warranted prior to clinical use. Additionally, it was shown that patients with high ABCG2 immunoreactivity could be candidates for specific treatment consisting of ABCG2 inhibition and irinotecan.

Regorafenib is a drug therapy administered to patients diagnosed with chemo-refractory mCRC and targets a wide range of receptor tyrosine kinases (RTKs). In the current Special Issue, an interesting study conducted by Raimondi et al. is presented in the context of resistance to this treatment. Considering the correlation between PD 1/PD-L1 checkpoint pathway and RTK inhibition–which has been examined in several tumor types– PD-L1 expression on circulating tumor cells (CTCs) was investigated in patients treated with regorafenib as third-line treatment [5]. It was revealed that PD-L1 expression in CTCs could predict the responsiveness of patients to regorafenib treatment, offering highly individualized treatment options. Moreover, CTCs emerged as a real-time, non-invasive biopsy procedure, able to evaluate PD-L1 expression in mCRC patients treated with regorafenib.

Based on the great potential of CTCs as a non-invasive prognostic or diagnostic approach, another interesting study, conducted by Hamid et al., investigated the genetic heterogeneities in CRC patients’ CTCs [6]. The genetic profiling of single CTCs revealed the heterogenous expression of particular genes among CTCs from different patients but in the same individual, as well. Therefore, single-cell genetic analysis could be used for monitoring the genetic heterogeneity, as well as for selection of a personalized therapeutic approach in clinical practice. The latter paves the way for further investigation regarding the potential exploitation of CTCs in clinical practice.

As aforementioned, besides metastasis, the poor prognosis of high-grade tumors constitutes a great challenge. The current TNM staging system is inefficient to predict the outcome of TNM stage II and III CRC patients and therefore, there is an urgent need for the identification of novel biomarkers to better stratify these patients. Towards this direction, Artemaki et al. examined the prognostic value of the *L*-DOPA decarboxylase (*DDC*) gene in TNM stage II and III CRC patients [7]. Interestingly, the potential value of *DDC* novel exons rather than complete mRNA transcripts was evaluated, leading to the conclusion that specific novel exons could be used as indicators for CRC patients’ survival and be able to stratify CRC TNM stage II and III patients into subgroups with distinct survival. Additionally, the significance of deregulated alternative splicing towards the generation of cancer specific transcripts emerged in this research study.

A great proportion of CRC cases have a family history of CRC; however, a small percentage of these cases is characterized by a well-established hereditary disorder, and the majority of them most likely has a complex multigenetic cause. Therefore, it is important to thoroughly examine this heritability, since a genetic diagnosis may favor patients’ prognosis, surveillance, and counseling. Additionally, it could assist in disease prevention for family members. Despite numerous high-throughput sequencing studies, a low number of genes associated with hereditary CRC and polyposis have been identified in the past decade. In this context, te Paske et al. conducted a research study which reviewed the design of studies having used whole genome/exome sequencing for the identification of genetic mutations which are critically involved in hereditary CRC and polyposis. Moreover, recommendations were provided regarding the optimization of the discovery and validation strategies [8]. The study concluded that the combined approach of a phenotype-driven, tumor-based candidate gene search can enlighten the potential contribution of novel genetic predispositions in genetically unknown hereditary CRC and polyposis.

In addition, several studies have identified genetic polymorphisms associated with CRC prognosis. During the last few years, extensive efforts have been undertaken to map tissue-specific regulatory variants of the human genome, concluding to a wide range of tools and databases which expedite the functional characterization of polymorphisms. The research study of Deutelmoser et al. illustrates the challenges of the assessment of gene expression in the context of CRC prognosis, based on individual genotype data. Moreover, it underlines the importance in assessing the prediction accuracy through measuring gene expression in a subset of the investigated study population. The implementation of this procedure led to the identification of ARID3B as a potential modifier of obese CRC patients’ survival [9]. Additionally, polymorphisms in angiogenesis-related genes potentially predispose to CRC or affect survival of CRC patients. Scherer et al. points to novel evidence regarding the impact of angiogenesis-related genetic variants on CRC outcome, uncovering the importance of *EFNB2*, *MMP2*, and *JAG1* genes, which are well-known for their functional role in angiogenesis [10].

The finding regarding polymorphisms in angiogenesis-related genes is quite significant, considering the key role of angiogenesis in CRC progression. Angiogenesis is a tightly regulated process, mediated by a group of angiogenic factors such as vascular endothelial growth factors (VEGFs) and their receptors [11]. Particularly, circulating levels of VEGF family members are associated with CRC patients’ survival [12]. Besides the direct role of VEGF signaling pathway in angiogenesis and cancer progression, proteases have been reported to exert a critical role in this process, as well. For instance, tryptase is secreted by mast cells and its activation leads to cell proliferation and release of pro-angiogenic factors. Moreover, tryptase degrades extracellular matrix components, promoting the invasion and metastasis of tumor cells [13]. Interestingly, recent studies uncovered that its high expression levels in serum of CRC patients are associated with a poor prognostic outcome of these patients [14]. These findings are quite interesting and support the biomarker utility and therapeutic targeting of molecules involved in angiogenesis.

A formerly neglected RNA type, circRNAs, has arisen scientific interest due to their multifaceted implication in both physiological and pathological states. Particularly in CRC, circRNAs have been involved in both the development and progression of this malignancy, while they have emerged as potential biomarkers and therapeutic targets [15,16]. Karousi et al. identified the full exon structure of two novel circRNA transcripts of *BCL2L12*, an apoptosis-related gene that has been associated with carcinogenesis, implementing an interesting experimental approach of nested PCR with divergent primers. Additionally, their value as molecular biomarkers was assessed and the negative impact of the high expression levels of the one out of the two circRNAs on CRC patients’ overall survival was pointed out [17]. These findings support the continuously emerging significance of circRNAs in CRC and encourage further investigation.

Besides genetic abnormalities, mutations, and lifestyle, intestinal flora and microbiome play a decisive role in CRC initiation and progression. Fujiwara-Tani et al. investigated *Clostridium perfringens* enterotoxin (CPE) in the context of sessile serrated adenoma/polyp with dysplasia (SSA/P-D). SSA/P-D is an SSA/P with cellular dysplasia; patients with SSA/P-D entail an increased probability to develop CRC. Interestingly, it was uncovered that CPE could promote the malignant transformation of SSA/P-D by activating the YAP signaling pathway, designating CPE as a risk factor for CRC development. Even though these findings necessitate further research, the significance of regulating intestinal flora in the context of colorectal malignancies is highlighted [18].

All the aforementioned data underscore the multivariable development of CRC, the elevated crosstalk between pathogenesis mechanisms, and hence the fact that several parameters should be taken into consideration for the assessment of patients’ outcome. Besides the molecular and genetic background of CRC patients, patients’ immune response should be evaluated, as well. However, it is important to investigate the latter parameter in a wider context including, for instance, evaluation of the molecular background. The review article of Laghi et al. examines the biomarker utility of tumor-infiltrating lymphocytes in microsatellite-instable (MSI) and -stable tumors. It highlights that the high expression of this cell subpopulation is a favorable indicator for early-stage colon cancer, while its biomarker utility in advanced-stage colon cancer requires further investigation [19].

Overall, we believe that the present Special Issue contributes to the enlightenment of the current knowledge concerning potential molecular biomarkers in CRC, providing not only novel biomarker candidates, but also approaches for optimizing the strategies of discovery of these candidates. The great heterogeneity of CRC is illustrated in the variety of factors which are implicated in the initiation and progression of this malignancy. The microbiome, mutations in key genes for normal cell function, deregulated signaling pathways, deregulated alternative splicing, cancer-specific variants, and patients’ immune state play critical roles in CRC, while they also possess biomarker attributes, as supported by the aforementioned articles. Even though the current knowledge has been widened via the implementation of novel technologies, further investigation is essential for the establishment of a personalized approach.
